# Nonsynonymous amino acid changes in the α-chain of complement component 5 influence longitudinal susceptibility to *Plasmodium falciparum* infections and severe malarial anemia in kenyan children

**DOI:** 10.3389/fgene.2022.977810

**Published:** 2022-09-14

**Authors:** Evans Raballah, Kristen Wilding, Samuel B. Anyona, Elly O. Munde, Ivy Hurwitz, Clinton O. Onyango, Cyrus Ayieko, Christophe G. Lambert, Kristan A. Schneider, Philip D. Seidenberg, Collins Ouma, Benjamin H. McMahon, Qiuying Cheng, Douglas J. Perkins

**Affiliations:** ^1^ University of New Mexico-Kenya Global Health Programs, Kisumu, Kenya; ^2^ Department of Medical Laboratory Sciences, School of Public Health Biomedical Sciences and Technology, Masinde Muliro University of Science and Technology, Kakamega, Kenya; ^3^ Theoretical Biology and Biophysics Group, Theoretical Division, Los Alamos National Laboratory, Los Alamos, NM, United States; ^4^ Department of Medical Biochemistry, School of Medicine, Maseno University, Maseno, Kenya; ^5^ Department of Clinical Medicine, School of Health Sciences, Kirinyaga University, Kerugoya, Kenya; ^6^ University of New Mexico, Center for Global Health, Department of Internal Medicine, Albuquerque, NM, United States; ^7^ Department of Biomedical Sciences and Technology, School of Public Health and Community Development, Maseno University, Maseno, Kenya; ^8^ Department of Zoology, Maseno University, Maseno, Kenya; ^9^ Department of Applied Computer and Biosciences, University of Applied Sciences Mittweida, Mittweida, Germany; ^10^ University of New Mexico, Department of Emergency Medicine, Albuquerque, NM, United States

**Keywords:** severe malarial anaemia, complement 5, missense mutations, *P. facliparum*, malaria

## Abstract

**Background:** Severe malarial anemia (SMA; Hb < 5.0 g/dl) is a leading cause of childhood morbidity and mortality in holoendemic *Plasmodium falciparum* transmission regions such as western Kenya.

**Methods:** We investigated the relationship between two novel complement component 5 (C5) missense mutations [rs17216529:C>T, p(Val145Ile) and rs17610:C>T, p(Ser1310Asn)] and longitudinal outcomes of malaria in a cohort of Kenyan children (under 60 mos, n = 1,546). Molecular modeling was used to investigate the impact of the amino acid transitions on the C5 protein structure.

**Results:** Prediction of the wild-type and mutant C5 protein structures did not reveal major changes to the overall structure. However, based on the position of the variants, subtle differences could impact on the stability of C5b. The influence of the C5 genotypes/haplotypes on the number of malaria and SMA episodes over 36 months was determined by Poisson regression modeling. Genotypic analyses revealed that inheritance of the homozygous mutant (TT) for rs17216529:C>T enhanced the risk for both malaria (incidence rate ratio, IRR = 1.144, 95%CI: 1.059–1.236, *p* = 0.001) and SMA (IRR = 1.627, 95%CI: 1.201–2.204, *p* = 0.002). In the haplotypic model, carriers of TC had increased risk of malaria (IRR = 1.068, 95%CI: 1.017–1.122, *p* = 0.009), while carriers of both wild-type alleles (CC) were protected against SMA (IRR = 0.679, 95%CI: 0.542–0.850, *p* = 0.001).

**Conclusion:** Collectively, these findings show that the selected C5 missense mutations influence the longitudinal risk of malaria and SMA in immune-naïve children exposed to holoendemic *P. falciparum* transmission through a mechanism that remains to be defined.

## Introduction

Malaria remains a significant global public health challenge. Recent data from the World Health Organization (WHO) show that 241 million cases resulted in 627,000 malaria-related deaths globally (WHO, 2021). The African region had 228 million cases and 602,000 deaths, accounting for approximately 95% of the cases and 96% of the deaths globally. The majority of cases in the African region are due to *Plasmodium falciparum* infections, with 80% of the deaths occurring in children less than 5 years of age (WHO, 2021). In holoendemic *P. falciparum* transmission regions such as western Kenya, falciparum malaria is a leading cause of childhood morbidity and mortality (Perkins et al., 2011). Severe malaria in holoendemic regions primarily manifests as severe malaria anemia [SMA, hemoglobin (Hb) < 5.0 g/dl] in children less than 5 years of age who are yet to develop naturally-acquired immunity to malaria (Ong’echa et al., 2006; Obonyo et al., 2007).

The pathogenesis of SMA is complex and multifactorial, resulting from overlapping sequelae that include lysis of infected and uninfected erythrocytes, splenic sequestration of erythrocytes, and inefficient erythropoietic responses in bone marrow (Awandare et al., 2011; Perkins et al., 2011). Over the last 2 decades, studies by our team in western Kenya have demonstrated that dysregulation in the innate immune genes is central to the pathogenesis of SMA, and that polymorphic variability in such genes influences susceptibility to *P. falciparum* infections, SMA, and mortality (Ouma et al., 2008; Munde et al., 2017; Achieng et al., 2019; Anyona et al., 2019; Kisia et al., 2019).

An integral part of the innate immune response is the complement system, comprised of three activation pathways: classical, alternative, and lectin, all of which converge at complement component 3 (C3) protein (Ricklin et al., 2010). Cleavage of C3 results in two subunits: 1) C3a, an anaphylatoxin that elicits potent pro-inflammatory mediator and chemoattractant properties that facilitate clearance of opsonized pathogens by phagocytosis (Klos et al., 2009) and 2) C3b, which binds with C2b4b to form C5 convertase, a serine protease that cleaves C5 into C5a and C5b. C5a, like C3a, is an anaphylatoxin, while C5b binds to C6, C7, C8, and C9 to initiate formation of the membrane attack complex [MAC (C5b-C9)] (Müller-Eberhard, 1985; Hadders et al., 2012). Membrane insertion of the MAC causes lysis of the target cell and the release of pro-inflammatory mediators (Kilgore et al., 1996; Morgan, 2015).

Previous investigations revealed that the complement cascade is activated in children and adults during acute malaria (Greenwood and Brueton, 1974; Helegbe et al., 2007; Nyakoe et al., 2009). In addition, relative to aparasitemic children, those with malaria have elevated C3b deposition on red blood cells, a process that can enhance phagocytosis, and consequently, promote malarial anemia (Odhiambo et al., 2008). Mechanism-based *in-vitro* studies show that the *P. f*alciparum parasite can induce MAC formation through both the classical and alternative pathways (Roestenberg et al., 2007). Studies also show that protection against clinical malaria and high-density parasitemia is associated with acquired anti-malarial antibodies that inhibit *P. falciparum* invasion of erythrocytes, primarily through C1q fixation and activation of the classical pathway (Boyle et al., 2015). Recent longitudinal investigations by our group in Kenyan children also revealed that two missense mutations in the C3 gene [rs2230199 (2307C>G, Arg>Gly^102^) and rs11569534 (34420G>A, Gly>Asp^1224^)], altered susceptibility to both malaria and SMA during the developmental phase of naturally-acquired malarial immunity (Raballah et al., 2021).

To further characterize the role of complement in the pathogenesis of malaria and SMA, we explored two novel missense mutations in the C5 gene and its association with malaria, SMA and all-cause mortality. The human C5 gene consists of 43 exons and is located on chromosome 9q33.2 (Sherry et al., 1999). Inherited human deficiencies of C5, including [MAC (C5b-C9)] deficiency, are associated with recurrent infection by *Neisseria* species resulting in mild-to-moderate meningitis (Schejbel et al., 2013; Colobran et al., 2016). However, C5 deficient mice (C5^−/−^) are resistant to experimental cerebral malaria due to an inability to form the MAC, and as such, abrogate the immune response that drives pathogenesis (Colobran et al., 2016). Since the role of C5 mutations in human malaria pathogenesis has not been previously reported, we selected two missense mutations that had an elevated proportion of minor allele frequencies (MAF) in African populations relative to other variants that induce amino acid changes. The first variant, rs17216529:C>T, p. (Val145Ile), results in an amino acid change from valine to isoleucine at position 145, while the second variant, rs17610:C>T, p. (Ser1310Asn), causes a serine to asparagine transition at position 1,310. A previous study in a cohort of patients with symptomatic coronary artery disease, although underpowered, showed that carriers of the homozygous minor allele TT for rs17216529:C>T, p. (Ile145Ile) had a trend towards increased disease severity and bleeding events (Henes et al., 2021). However, an earlier study did not find any relationship between rs17216529:C>T and stroke in a cohort of patients with sickle cell disease (Flanagan et al., 2013). To date, there are no reports on the influence of rs17610:C>T on any human disease outcomes. Here, we present the impact of the two C5 missense mutations, and their combinations, on longitudinal susceptibility to malaria and SMA in a cohort of under- five Kenyan children residing in a holoendmic *P. falciparum* transmission region.

## Methods

### Study site and participants

Children (aged 2–48 months, n = 1,546) were enrolled at the Siaya County Referral Hospital (SCRH) in Siaya County, Kenya between April 2004 and September 2015 who had a confirmed positive blood smear for *P. falciparum* malaria. In addition, children were enrolled at SCRH who were receiving routine childhood vaccinations and had a negative blood smear for *P*. *falciparum* (i.e., aparasitemic controls). Siaya County is a rural region in western Kenya with holoendemic *P. falciparum* transmission whose inhabitants are primarily from the Luo ethnic group (>96%), hence the participants can be considered genetically similar (Githeko et al., 1994). Upon enrollment, comprehensive evaluations were performed on the study participants to collect clinical and demographic information, and information on the history of present illness. Children were excluded from study participation if they were non- *P*. *falciparum* positive on blood films, had prior hospitalizations (for any reason), underwent recent blood transfusions, and/or had evidence of cerebral malaria.

Study participants presenting with *P. falciparum* malaria (any density) were stratified according to hemoglobin concentrations into uncomplicated malaria (UM, Hb ≥ 5.0 g/dl) and SMA (Hb < 5.0 g/dl). All study participants also had HIV-1/2 and bacteremia status determined since our previous studies showed that these comorbidities influence the severity of malarial anemia (Otieno et al., 2006; Were et al., 2011). Parents/legal guardians of the children were provided with pre- and post-test HIV counselling. Required treatment interventions for clinical management followed the Ministry of Health (MOH)-Kenya guidelines. The study was approved by Maseno University Ethics Review Committee (MUERC; MSU/DRPI/MUERC/00510/18) and the University of New Mexico Institutional Review Board (16–284). Written informed consent was obtained from the parent/legal guardian of the study participants in the language of choice (English, Kiswahili, or Dholuo).

### Longitudinal follow-up

Parents/guardians were asked to return with their child every 3 months throughout the 36-months follow-up period (i.e. quarterly visit). If parents/guardians failed to return with their child for the scheduled quarterly visit, study personnel visited the residences to check on the health status of the children (including mortality). In addition, for proper clinical management and longitudinal documentation of childhood illnesses, parents/guardians were asked to bring their child to the hospital during any febrile episode(s) or other illnesses (i.e., acute visit). At each acute and quarterly visit, study participants underwent a complete physical and laboratory workup for proper clinical management (e.g., complete blood count, malaria parasitemia determination, evaluation of viral and bacterial infections, etc.). Throughout the follow-up period, mortality data were collected either from hospital records or by verbal autopsy in cases in which death occurred outside the hospital.

### Laboratory measures

Upon informed consent from the parents/legal guardians, approximately 3.0 ml venous whole blood samples were collected into EDTA-containing vacutainer tubes for the purposes of laboratory diagnostics, care and to conduct the experiments. *P*. *falciparum* density was determined following our previously published reports (Were et al., 2006). Additionally, complete blood counts were performed using a Beckman Coulter^®^ AcT diff2™ hematology analyzer (Beckman–Coulter Inc. Brea, CA, United States). The determination of sickle cell trait and sickle cell disease was performed using cellulose acetate electrophoresis according to the manufacturer’s standard operating procedures (Helena Bio-Sciences, Oxford, United Kingdom). It is however important to note that according to the manufacturer, this method has a limitatition in that, it may be difficult to discriminate between some abnormal hemoglobins, due to similarities in electrophoretic mobilities. Therefore, such cases must be distinguished using other available methods. Serological tests for HIV (Determine™ HIV-1/2, Abbott, Woodmead, South Africa and Uni-Gold™ HIV-1/2, Trinity Biotech Plc. Wicklow, Ireland) were used to determine HIV exposure. Proviral DNA PCR testing was performed for the participants that tested positive for HIV, with at least one the available serological tests, based on our previously published reports (Otieno et al., 2006). Bacterial comorbidity was assessed by culture and sensitivity assays following our previously published protocols (Were et al., 2011). Glucose six phosphate dehydrogenase (G6PD) deficiency (levels: normal, intermediate, deficient) was determined following the methods of Cherepnalkovski *et al.*, (Cherepnalkovski et al., 2015). The α^3.7^ deletion produces an HBA2/HBA1 hybrid gene and a 3.7 kb deletion (Borg et al., 2009). The α^3.7^− thalassemia deletion was determined according to published methods (Dode et al., 1993).

### C5 variant selection

Variants were selected by cataloging all known missense mutations reported for C5 with elevated MAFs (>10%) in African populations in the National Center for Biotechnology Information (NCBI) database (Sherry et al., 1999). This strategy was based on the rationale that low penetrance of variants in the population would likely not explain high rates of malaria in a region with long-standing holoendemic *P. falciparum* transmission. Allelic distributions of C5 candidate variants were determined for the following populations: global, African (AFR), Luhya (LWK), and Maasai (MWK) using data from the International HapMap and 1000 Genomes Projects. Additional criteria included selecting variants that were in linkage equilibrium, i.e., not strongly co-inherited (Machiela and Chanock, 2015). Based on the above criteria, two missense mutations were selected: 1) rs17216529:C>T - valine to isoleucine at amino acid position 145 p. (Val145Ile), and 2) rs17610:C>T - serine to asparagine at position 1,310 p. (Ser1310Asn). Analysis using the GRCh38 high coverage genome build for the 1000 Genomes Project (Luhya, Kenya, LWK) revealed that the two SNPs were not linked, i.e., in linkage equilibrium (D’ = 0.763; *R* ^2^ = 0.021; χ^2^ = 4.226; *p* = 0.040] (Machiela and Chanock, 2015). Neither of the selected C5 variants, nor their combinations, have been explored in malaria.

### Visualization of the C5 variants on 3-dimensional (3D) protein structures

The reference sequence for the human C5 protein was retrieved from Genbank at the NCBI with the accession number NP_001304092. The potential impact of the C5 SNPs on the overall protein structure was investigated using AlphaFold (Jumper et al., 2021; Varadi et al., 2022) with 3D protein structures visualized using UCSF Chimera (Pettersen et al., 2004). The wild-type (WT) 3D protein structure for C5 was previously determined by Fredslund *et al.*, at 3.1 Å resolution using X-ray diffraction (Fredslund et al., 2008). The structure is deposited as code 3CU7 in the Protein Data Bank (PDB). The WT structure for the membrane attack complex, including C5b, was previously determined by Menny *et al.,* at 5.6 Å using electron microscopy and deposited as 6h04 in the PDB (Menny et al., 2018).

### Genotyping of C5 variants

Prior to performing the genotyping assays, genomic DNA was extracted from cheek cells (WBCs) collected on buccal swabs using the MasterAmp™ Buccal swab DNA Extraction kit (Epicentre Biotechnologies, Madison, WI, USA). Further, the quality of the isolated DNA was assessed using a Qubit 3.0 Fluorometer (Thermofisher Scientific, Waltham, MA, United States). High-quality DNA samples were amplified using the GenomiPhi^®^ system (GE Healthcare, South Plainfield, NJ, United States) to obtain sufficient DNA quantities for the genotyping assays. TaqMan^®^ 5′ allelic discrimination Assay-By-Design high-throughput method was used to genotype the C5 rs17216529:C>T (Assay ID: C_32672495_10) and rs17610:C>T (C_11720403_20) variants, following the manufacturer’s protocols (Thermofisher Scientific, Carlsbad, CA, USA), using a StepOne Plus™ Real-Time PCR System. The PCR reaction was performed in a total reaction volume of 10.0 μl with the following amplification cycles: initial denaturation (60°C for 30 s and 95°C for 10 min) followed by 40 cycles of (95°C for 15 s and 60°C for 1 min) and a final extension (60°C for 30 s) using allele-specific fluorescence. The StepOne™ Software (Version 2.3) was used for allelic discrimination (Thermofisher Scientific, Carlsbad, CA, United States).

### Statistical analyses

The demographic, clinical, and laboratory characteristics collected at enrollment were analyzed using SPSS, version 23.0 (IBM SPSS Statistics Inc. Chicago, IL, United States). Across group comparisons were determined using Kruskal–Wallis tests, and when significant, pairwise comparisons were performed using the Mann-Whitney *U* test. Proportions across the groups were compared using Chi-square tests. Hardy-Weinberg equilibrium (HWE) was determined by the exact test (Schaid et al., 2006). We tested whether the number of malaria episodes, SMA episodes, and mortality was proportional to the genotype frequencies at the two SNPs using chi-square goodness-of-fit tests. *p*-values ≤ 0.05 were considered statistically significant.

Linkage disequilibrium (LD) for the cohort was determined by Multiallelic Interallelic Disequilibrium Analysis (MIDAS) software version 1.0 (Gaunt et al., 2006). The association between the C5 genotypes/haplotypes on the longitudinal episodes of malaria, SMA, and mortality were determined using R (version 3.1.3) (Team, 2019). Whenever necessary, we performed secondary analyses using the additive model, that discriminates the presence of either one or two copies of a haplotype. Phasing of the haplotypes was performed using R. The association between C5 variants and the frequency of malaria and SMA episodes was determined by a Poisson rate regression (R glm function, family = Poisson), with the (logarithm of) age at the patients’ last visits being the offset variable (rate regression). A model search based on minimizing the AIC, using a forward-backward selection was performed with C5 genotypes/haplotypes as covariates, along with additional covariates that can influence anemia: age at enrollment, sex, HIV status (unexposed uninfected, exposed-uninfected, and positive), cohort, sickle cell status (sickle trait and sickle cell disease), G6PD deficiency and α^3.7^− thalassemia status. In addition, Multiple-event per subject Cox model (Anderson Gill Ref) (R survival package version 2.38.2, coxph function) was performed to delineate the relationship between C5 variants and all-cause mortality (death during the follow-up period, including malaria-unrelated causes of mortality) using identical covariates as the Poisson models and a forward-backward selection. For each model, Bonferroni-Holm corrections were performed to adjust for multiple comparisons.

## Results

### Characteristics upon enrollment

The study included a total of 1,546 study participants who were stratified into three categories at enrollment: aparasitemic (n = 288), uncomplicated malaria (UM, Hb ≥ 5.0 g/dl, n = 971) and SMA (Hb < 5.0 g/dl, n = 287). Demographic, clinical, and laboratory characteristics of the study participants upon entry into the study are presented in Table 1. The distribution of males and females was comparable across the groups (*p* = 0.898). However, age differed across the three groups (*p* = 4.480 × 10^−4^), with the SMA group being the youngest.

**TABLE 1:** Demographic, clinical, and laboratory characteristics of study participants at enrollment.

Characteristics	Aparasitemic	UM (Hb ≥ 5.0 g/dl)	SMA (Hb < 5.0 g/dl)	*P*
**Demographic parameters**			
Sample size (n)	288	971	287	
Sex, n (%)				
Female	145 (50.35)	480 (49.43)	146 (50.87)	0.898^a^
Male	143 (49.65)	491 (50.57)	141 (49.13)	
Age, (months)	11.16 (13.15)	12.77 (10.43)	9.83 (10.60)**	**4.480** × **10** ^**−4**^ ^b^
**Hematological indices and parasitemia**			
Hematocrit, (Hct. %)	32.90 (7.40)	25.30 (9.00)	14.30 (3.80)**	**8.543** × **10** ^**−141**^ ^b^
Hemoglobin, g/dL	10.35 (2.70)	7.70 (2.90)	4.30 (1.20)**	**4.202** × **10** ^**−164**^ ^b^
RBC, (×10^12^/μl)	4.67 (1.12)	3.79 (1.41)	1.91 (0.69)**	**6.420** × **10** ^**−131**^ ^b^
MCV	70.00 (10.40)	69.00 (11.05)	73.00 (13.73)**	**1.986** × **10** ^**−11**^ ^b^
MCH	22.20 (3.90)	21.30 (4.10)	21.90 (4.30)**	**5.617** × **10** ^**−7**^ ^b^
MCHC	31.70 (2.70)	30.70 (2.60)	30.15 (4.30)*	**1.862** × **10** ^**−10**^ ^b^
RDW	18.60 (5.50)	20.30 (4.60)	22.75 (5.80)**	**4.642** × **10** ^**−27**^ ^b^
WBC, (×10^3^/μl)	11.00 (7.20)	11.70 (6.60)	14.30 (9.40)**	**1.085** × **10** ^**−7**^ ^b^
Monocytes, (×10^3^/μl)	7.70 (4.30)	7.90 (5.30)	9.15 (7.00)**	**4.823** × **10** ^**−8**^ ^b^
Granulocytes, (×10^3^/μl)	36.00 (20.00)	45.20 (25.10)	39.30 (21.10)**	**3.364** × **10** ^**−16**^ ^b^
Lymphocytes, (×10^3^/μl)	55.80 (18.40)	46.15 (21.20)	49.90 (17.00)*	**2.239** × **10** ^**−18**^ ^b^
Platelet Counts, (×10^3^/μl)	341.00 (224.00)	152.00 (123.80)	141.50 (92.50)	**1.048** × **10** ^−66^ ^b^
Parasite density/μl	0.00 (0.00)	28,844.70 (79,216)	24,200.00 (69,659)*	**0.032** ^c^
**Genetic variants**			
Sickle Cell trait, n (%)				
HbAA	221 (78.65)	799 (83.14)	254 (91.04)*	
HbAS	51 (18.15)	158 (16.44)	19 (6.81)*	**7.113** × **10** ^−**7**^ ^a^
HbSS	9 (3.20)	4 (0.42)	6 (2.15)*	
G6PD				
Normal	180 (62.50)	628 (64.68)	200 (69.69)	
Intermediate	47 (16.32)	173 (17.82)	36 (12.54)	0.294^a^
Deficient	11 (3.82)	36 (3.71)	13 (4.53)	
α^3.7^-Thal deletion, n (%)				
αα/αα	97 (33.68)	338 (34.81)	94 (32.75)	
α^3.7^/αα	69 (23.96)	310 (31.93)	97 (21.53)	**0.025^a^**
α^3.7^/αα	62 (21.53)	148 (15.24)	47 (16.38)
**Co-infections**			
Bacteremia, n (%)	25 (8.68)	58 (5.97)	26 (9.06)	0.067^a^
HIV-1, n (%)	12 (4.17)	28 (2.88)	24 (8.36)*	**2.250x10** ^**−4**^ ^a^

Study participants (n = 1,546) were stratified into three groups, aparasitemic, SMA (i.e., Hb < 5.0 g/dl with any density parasitemia) or UM (Hb ≥ 5.0 g/dl with any density parasitemia). Data presented are medians (interquartile range, IQR), unless otherwise stated.

a Statistical significance determined by Fisher’s exact test.

b Differences were determined using Kruskal–Wallis tests, and where significant differences were observed, pairwise comparisons between UM and SMA group were performed using Mann-Whitney U tests. Bold values indicates a *p* value of ≤0.050.

*Represents significant pairwise comparisons between UM and SMA *p*-value < 0.050, and ** represents *p*-value < 0.001.

c Differences between UM and SMA group were performed using Mann-Whitney *U* tests and Fisher’s exact test (one-sided for Bacteremia and HIV-1, two-sided unless stated otherwise). NB: The percentages for sex and sickle cell trait are worked down the column, while those of bacteremia and HIV-1 are calculated across the rows.

^*^RBCs = red blood cells; MCV = mean corpuscular volume; MCH = mean corpuscular hemoglobin; MCHCH = mean corpuscular hemoglobin concentration; RDW = red blood cell distribution width and WBCs = white blood cells; Hb AA = hemoglobin AA; Hb AS = Hemoglobin AS; Hb SS = Hemoglobin SS; α-Thal = α-thalassemia 3.7 kb deletion; G6PD = glucose 6 phosphate dehydrogenase.

Bold indicate significant *p* values.

Hematocrit (Hct), Hemoglobin (Hb) concentrations, and red blood cell (RBC) counts progressively declined across the three groups (*p* = 8.543x10^−141^, *p* = 4.202 × 10^−164^, *p* = 6.420 × 10^−131^, respectively) and were lowest in children with SMA, as expected based on *a priori* grouping. Mean corpuscular volume (MCV) was elevated in children with SMA and differed across the groups (*p* = 1.986 × 10^−11^), while mean corpuscular hemoglobin (MCH) and mean corpuscular hemoglobin concentration (MCHC) were highest in the aparasitemic group with an overall group difference (*p* = 5.617 × 10^−7^ and *p* = 1.862 × 10^−10^, respectively). The red blood cell distribution (RDW) and counts for white blood cells (WBCs) and monocytes varied in the cohort (*p* = 4.642x10^−27^, *p* = 1.085 × 10^−7^ and *p* = 4.823 × 10^−8^, respectively) and were highest in children with SMA. Granulocyte counts also varied (3.364 × 10^−16^) with the UM group having the highest values. Lymphocyte counts differed in the cohort (*p* = 2.239 × 10^−18^) and were highest in aparasitemic children, while platelet counts progressively decreased across the groups (*p* = 1.048 × 10^−66^) and were lowest in children with SMA. Parasite density in peripheral blood was lower in children with SMA relative to those with milder forms of malaria (UM, *p* = 0.032).

Analysis of hemoglobin S (HbS) carriage revealed that the frequency of HbAS (protective alleles) was highest in aparasitemic children and lowest in those presenting with SMA (*p* = 7.113 × 10^−7^). The proportion of G6PD variants was comparable across the groups (*p* = 0.294), while the proportion of thalassemia variants differed across the groups (*p* = 0.025). In addition, results from our models show that both HbAS and HbSS reduced the risk of malaria episoded [incident risk ratio (IRR) = 0.799, 95%CI: 0.730–0.855, *p* = 4.666 × 10^−9^ and IRR = 0.528, 95%CI: 0.388–0.718, *p* = 4.796 × 10^−5^, respectuvely). Similarly, homozygous mutants (*α*/α) for thallassemia reduced malaria during the study period (IRR = 0.900, 95%CI: 0.837–0.967, *p* = 0.004). However, after acquisistion of malaria only HbAS and HbSS impacted on the development of SMA. Carriage of HbAS reduced the risk of SMA (IRR = 0.499, 95%CI: 0.334–0.746, *p* = 7.042 × 10^−4^), while inheritance of HbSS increased the risk of SMA 3-fold (IRR = 3.108, 95%CI: 1.771–5.455, *p* = 7.77x10^−5^). The proportions of these genetic variants in the study population relative to the frequencies of malaria and SMA epidosodes are shown in Supplementary Table S2. Since our previous investigations demonstrated that bacteremia and HIV exacerbate the development of SMA (Otieno et al., 2006; Were et al., 2011), the absence/presence of these co-infections was determined in the study population. The proportion of children with bacteremia did not significantly differ between the groups (*p* = 0.067). However, the proportion of HIV infection differed across the groups (*p* = 2.250x10^−4^) and was highest in children with SMA. As expected based on *a priori* grouping, the RBC indices i. e RBC counts, Hct and Hb were strongly correlated (Supplementary Figure S1). These results are consistent with our previous findings and underscore the importance of considering age, carriage of HbS, and co-infection status in the complex phenotype of SMA (Davenport et al., 2010; Novelli et al., 2010; Were et al., 2011).

### Modeling of the C5 protein structure

**FIGURE 1:**
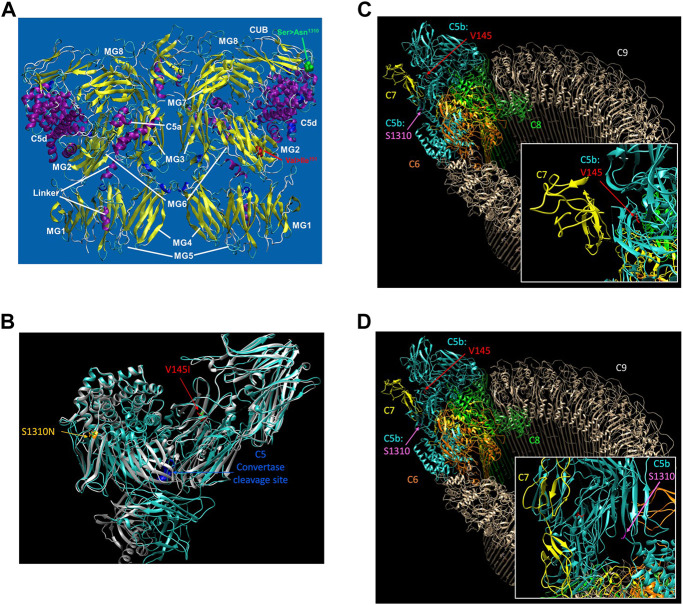
The reference sequence for the human C5 protein was retrieved from Genbank at the NCBI with the accession number NP_001304092. The potential impact of the C5 SNPs on the overall protein structure were investigated using AlphaFold (Varadi et al., 2022) with 3D protein structures visualized using UCSF Chimera (Pettersen et al., 2004) **(A)**. The structure of C5 showing the various domains i.e., macroglobulin (MG)1-MG8. Relative to the published C5 structure (Fredslund et al., 2008), we observed that Val145le mutation is located in the macroglobulin MG2 domain, while the Ser1310Asn is located on the CUB **(B)**. AlphaFold structure prediction of rs17216529:C>T and rs17610:C>T. Inspection of the wild-type (WT) and mutant proteins revealed no significant perturbations to the overall structure. However, subtle changes that may not be detected by AlphaFold may impact on the unstable C5b. The WT molecule for both V145I and S1310N are colored green, while the mutant for V145I is colored red and that of S1310N is colored orange **(C)**. AlphaFold structure prediction of the two SNPs relative to the cleavage site for C5 convertase. Examination of the structure revealed that the SNPs are also distal from the C5 convertase cleavage site. Additionally, the WT structure of both C5 and C5b in the MAC, rs17216529:C>T appeared to be buried, orienting the hydrophobic side chain into the protein. On the other hand, we did not observe a clear interaction partner for rs17216529:C>T neither in the C5 nor MAC structures. However, the mutation is located near the C5b and C7 in the MAC structure **(D)**. AlphaFold structure prediction of the location of mutation on rs17610:C>T. Examination of the C5 protein structure revealed that the mutation is exposed both on the C5 protein, and in the MAC.

The structure of the C5 protein is shown in Figure 1A. Analysis of the protein structure revealed that the amino acid transitions from the missense mutations were in the *α*−chain. rs17216529:C>T, p. (Val145Ile) lies within the macroglobulin 2 (MG2) domain, while rs17610:C>T, p. (Ser1310Asn) is in the CUB domain (for complement C1r/C1s, Uegf, Bmp1). The Val to Ile transition at position 145 does not change the amino acid charge since both Val and Ile are non-polar. Similarly, the Ser to Asn transition at position 1,310 does not impart any changes in amino acid charge since Ser and Asn are both polar residues.

Accordingly, AlphaFold structure prediction of both rs17216529:C>T, p. (Val145Ile) and rs17610:C>T, p. (Ser1310Asn) revealed no significant perturbations to the overall structure between the WT and mutant proteins **(** Figure 1B). However, the mutation does lie close to the interface between C5b and C7 in the MAC structure. Therefore, the mutation may still impact C7 binding through subtle perturbations e.g. through changes to the stability of the local structure (Pak et al., 2021) and/or changes in aggregation potential, as is the case with sickle cell disease (Lu et al., 2019) as current modeling techniques may not adequately capture these impacts (Pak et al., 2021). Both sites are also distal from the C5 convertase cleavage site. In the WT structure of both C5 and C5b in the MAC, rs17216529:C>T, p. (Val145Ile) is buried, with the hydrophobic side chain oriented into the interior of the protein (Figure 1C). No clear interaction partner of rs17216529:C>T, p. (Val145Ile) is evident in either the C5 or MAC structures. However, the mutation does lie close to the interface between C5b and C7 in the MAC structure (Figure 1C), so subtle structural perturbations, not anticipated by AlphaFold, could impact C7 binding. In contrast, the rs17610:C>T, p. (Ser1310Asn) mutation is located on the surface of both the C5 protein, and in the MAC (Figure 1D). The Ser in the WT structure is not located at an interface with any other components of the MAC, suggesting minimal impacts on the MAC structure.

### Location and allelic distribution of the C5 SNPs

**FIGURE 2:**
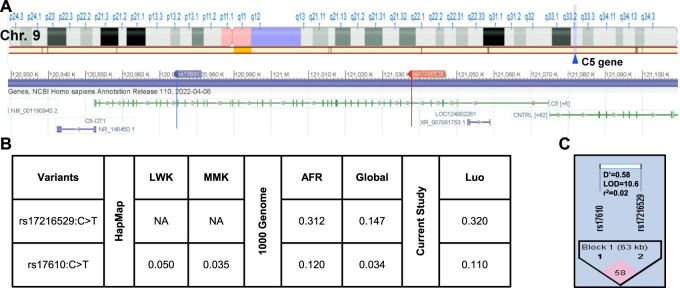
C5 chromosome location and linkage disequilibrium (LD) plot of the selected missense mutations **(A).** Location of C5 on Chromosome 9q33.2. C5 consists of 1,682 amino acids and consists of 43 exons. Chromosome position (build GRCh38. p14) for the investigated SNPs rs17216529:C>T and rs17610:C>T. The alignment and gene structure for C5 was retrieved from dbSNP database **(B).** C5 genotype minor allele frequencies (MAF) from the HapMap database for Kenyan populations [Luhya (LWK), Maasai (MKK) and calculated by MIDAS for the Luo (current study). MAFs from the 1,000 Genome project shows the African (AFR) and global allele frequencies. MAFs for the current Luo (Kenyan) population are also presented **(C).** Linkage disequilibrium plot for rs17216529:C>T and rs17610:C>T SNPs (D’ = 0.581, LOD = 10.63, *R* ^2^ = 0.02). The two SNPs are in linkage equilibrium, that is, they are inherited independently.

The location of the two SNPs in the C5 gene at q33.2 of chromosome nine is shown in Figure 2A. As discussed above, the selection strategy for choosing C5 variants included prioritizing those with elevated MAFs in African populations. The MAFs for rs17216529:C>T and rs17610:C>T from the International HapMap Project, 1,000 Genome Project, and genotyping of the Kenyan cohort (Figure 2B) (Sherry et al., 1999; International Hap Map, 2003). Relative to the MAF in the global population (0.147), rs17216529:C>T is enriched in AFR (0.312), and slightly higher in the cohort studied here (Luo, 0.320). The MAF for rs17610:C>T is also higher in AFR (0.120) than in the global population (0.034) which is comparable to the LWK (0.050) and MMK (0.035). Thus, the MAF for the Kenyan cohort (Luo. 0.110) is comparable to AFR (0.120) and 3-fold higher than the global population (0.034). Consistent with the linkage results in the LWK population used to guide variant selection, MIDAS revealed that rs17216529:C>T and rs17610:C>T were also in linkage equilibrium (i.e., not considered linked) in the current cohort (D’ = 0.581, LOD = 10.63, *R* ^2=^0.02, Figure 2C). The distribution of genotypes and haplotypes for rs17216529:C>T, p. (Val145Ile) and rs17610:C>T, p. (Ser1310Asn), and HWE analyses are presented in Supplementary Table S1. Collectively, these results show the selected C5 variants are enriched in the Luo population and not strongly co-inherited.

### Influence of C5 variants on *P. falciparum* infections, SMA, and all-cause mortality

Prior to the development of adaptive immunity, children in holoendemic *P. falciparum* transmission regions experience many episodes of malaria. As such, longitudinal studies offer robust platforms to investigate the influence of genetic variants on susceptibility to disease outcomes. The impact of the C5 variants on susceptibility to malaria, SMA, and mortality were determined throughout a 36-month period coinciding with the development of naturally-acquired malarial immunity. As shown in Table 2 (stratified according to genotype/haplotype), there was a high number of both malaria and SMA episodes in the cohort across the follow-up period, and a total of 99 deaths.

**TABLE 2:** Number of malaria and SMA episodes, and all-cause mortality across the 36-months follow-up period stratified by genotype/haplotype.

Genotype/Haplotype	Amino acid	Malaria episodes	SMA episodes	Mortality
**rs17216529:C>T**	**Val151Ile**	Total = 6,892	Total = 382	Total = 81
CC	Val151Val	3,245	173	40
CT	Val151Ile	2,644	140	33
TT	Ile/Ile^**151**^	1,003 (14.55)	69	8
χ^2^ goodness of fit test		χ^2^ = 78.754	χ^2^ = 0.711	χ^2^ = 2.562
		***p*<0.001**	*p* = 0.701	*p* = 0.278
**rs17610:C>T**	**Ser1310Asn**	Total = 7,173	Total = 405	Total = 82
CC	Ser1310Ser	6,231	341	70
CT	Ser1310Asn	490	35	7
TT	Asn1310Asn	452 (6.30)	29	5
χ^2^goodness of fit test		χ^2^ = 17.981	χ^2^ = 2.196	χ^2^ = 0.704
		***p* = 0.00012**	*p* = 0.334	*p* = 0.703
**rs17216529:C>T/rs17610:C>T**	**Val151Ile/Ser1310Asn**	Total = 7,758	Total = 447	Total = 99
Non-CC	non-Val151Ser1310	1,596	113	16
CC	Val151Ser1310	6,162	334	83
Non-CT	non-Val151Asn1310	4,259	247	61
CT	Val151Asn1310	3,499	200	38
Non-TC	non-Ile151Ser1310	6,797	384	87
TC	Ile151Ser1310	961	63	12
Non-TT	non-Ile151Asn1310	7,604	437	96
TT	Ile151Asn1310	154	10	3

Data are presented as numbers of malaria, SMA and mortality cases during the 36-months follow-up period. These were classified by the C5 genotypes and haplotypes. The summary statistics were performed using R (version 3.1.3).

Bold indicate significant *p* values.

Non-CC refers to non-wild genotypes for both SNPs, consistently non-CT is non-C in the first SNP and non-T in the second SNP.

The number of malaria and SMA episodes, as well as mortality cases were recorded throughout the 36-months follow-up period (Table 2). A total of 6,892 malaria cases were reported for rs17216529:C>T. Children with the wild-type CC genotype had the highest number of malaria cases (3,245), followed by the heterozygous CT (2,644), and homozygous mutant TT (1,003) carriers. Cases of malaria for rs17610:C>T, 7,173 were distributed as follows: wild-type CC (6,231), heterozygous CT (490), and homozygous mutant (452). In addition, during the study period, 382 cases of SMA were observed for rs17216529:C>T. The distribution of SMA cases for rs17610:C>T were: wild-type CC (173), heterozygous CT (140) and homozygous mutant TT 69) SMA cases. Finally, mortality cases were recorded for both SNPs during the follow-up period. There were 81 deaths recorded for rs17216529:C>T, distributed into wild-type CC (40), heterozygous CT (33) and homozygous mutant TT (8). For rs17610:C>T, there were 82 cases of mortality within the 36-months study period, categorized into wild-type CC (70), heterozygous CT (7), and homozygous mutant TT (5).

The cases of malaria, SMA, and mortality were also assessed in the haplotypic combinations. There were 7,758 malaria cases for all the haplotypes. These were stratified into CC (6,162), CT (3,499), TC (961), and TT (154). The SMA cases were 447 for all the haplotypes and distributed into CC (334), CT (200), TC (63), and TT (10). Lastly, 99 mortality cases were reported across the haplotypes. The following deaths occurred in CC (83), CT (38), TC (12), and TT (3). Additionally, we performed chi-square goodness of fit test to determine if the number of malaria, SMA and death were elevated that expected in the mutant cases. These analyses revealed that the number of malaria episodes among mutant homozygotes at rs17216529:C>T were higher than expected (Table 2), but lower in the mutant homozygotes at rs17610:C>T. The distribution of SMA episodes and mortality did not differ from the genotype distribution for both SNPs. However, due to the relatively low cases of SMA and mortality there is not much discriminative power at this aggregate level.

**FIGURE 3:**
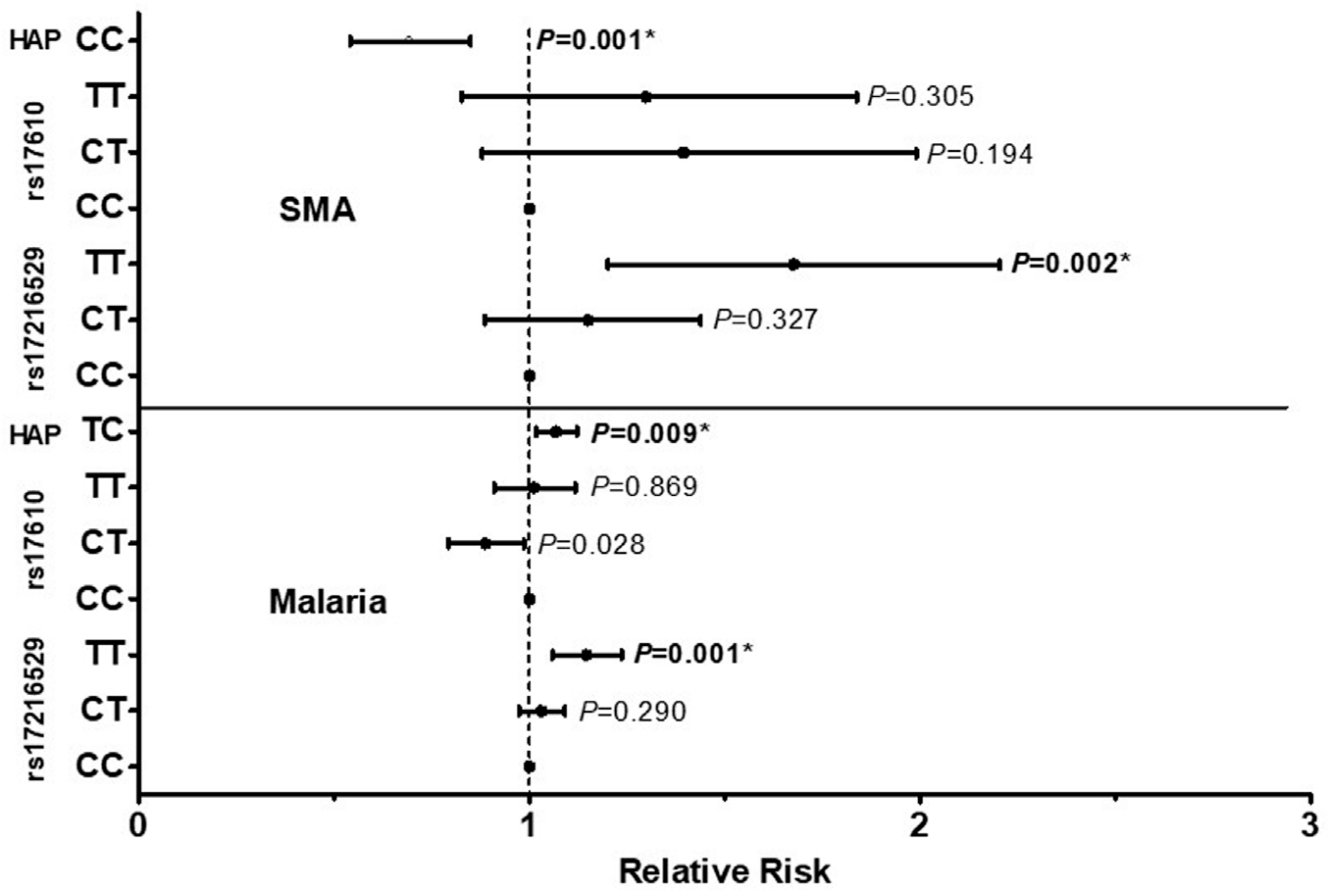
A total of 1,546 study participants were enrolled into the study. These were grouped into three categories at enrollment: aparasitemic (n = 288), uncomplicated malaria (UM, Hb ≥ 5.0 g/dl, n = 971) and SMA (Hb < 5.0 g/dl, n = 287). The influence of C5 genotypes/haplotypes on the number of malaria and SMA episodes throughout the study period was determined by a Poisson rate regression (R glm function, family = Poisson), with the (logarithm of) age at the patients last visits being the offset variable (rate regression). A forward-backward model selection that minimize the AIC was used in the Poisson regression. The first covariate inputs into the model were either C5 genotypes or haplotypes. Thereafter, additional covariates which included age at enrollment, sex, HIV, cohort, sickle cell status (sickle trait and sickle cell disease) glucose six phosphate deficiency (G6PD) and α thalassemia were entered into the model. The figure shows relative risk (midline dot) and 95% confidence intervals as determined by log-linear regression analyses, adjusting for age at enrollment, sex, HIV, cohort and sickle cell status (sickle trait and sickle cell disease). This cohort for followed quarterly for a period of 36 months *Significant after Bonferroni-Holm correction.

The influence of the C5 variant genotypes and haplotypes on susceptibility to malaria and SMA is shown in Figure 3. Genotypic analyses revealed that homozygous carriage of the mutant allele (TT) for rs17216529:C>T, p. (Ile145Ile) increased the risk of both malaria [incident risk ratio (IRR) = 1.144, 95%CI: 1.059–1.236, *p* = 0.001) and SMA (IRR = 1.627, 95%CI: 1.201–2.204, *p* = 0.002], with both results remaining significant after adjusting for multiple comparisons. The genotypic model also showed that heterozygous carriers (CT) for the rs17610:C>T, p. (Ser1310Asn) variant had a reduced risk of malaria (IRR = 0.885, 95%CI: 0.794–0.987, *p* = 0.028], however, the results did not remain significant after correction for multiple comparisons. None of the genotypic combinations of rs17610:C>T significantly altered susceptibility to SMA.

Further analyses exploring the impact of haplotypic combinations on susceptibility to malaria and SMA revealed that carriers of the TC p.(Ile145Asn1310) haplotype had an increased risk of malaria (IRR = 1.068, 95%CI: 1.017–1.122, *p* = 0.009, Bonferroni-Holm significant), while inheritance of both wild-type alleles (CC, Val145Ser1310) reduced the risk of SMA (IRR = 0.679, 95%CI: 0.542–0.850, *p* = 0.001, Bonferroni-Holm significant). Although only results that emerged from the forward-backward selection were reported for the haplotypic models, we performed secondary analyses by forcing the TC haplotype into a binary and additive model for SMA. Carriage of the TC haplotype non-significantly increased the risk of SMA in the binary model (IRR = 1.187, 95%CI: 0.968–1.454, *p* = 0.098), and significantly increased the risk of SMA in the additive model (IRR = 1.192, 95%CI: 1.038–1.368, *p* = 0.013). Thus, it appears that inheritance of the T allele(s) at rs17216529:C>T has a substantial impact on both *P. falciparum* infections and the development of SMA. None of the other haplotypic combinations emerged in the selection models for influencing susceptibility to either malaria or SMA. Moreover, neither the genotypes nor the haplotypes investigated influenced susceptibility to all-cause mortality in the Cox regression analyses.

## Discussion

In holoendemic transmission regions for *P*. *falciparum* such as western Kenya, SMA has remained a leading cause of morbidity and mortality in immune naïve children. Previous studies in experimental human malaria have shown that complement activation is an early immune response marker against *P. falciparum* infections (Roestenberg et al., 2007). The cleavage of C5 by C5 convertase is critical for activation of the terminal complement pathway which forms the MAC (C5b-C6-C7-C8-C9) on target pathogens, resulting in cell lysis and apoptosis (Merle et al., 2015). Previous investigations demonstrated that there are elevated levels of soluble C5b-C9 in falciparum malaria (Wenisch et al., 1997). Prior studies also demonstrated C5-dependent *P*. *falciparum* sporozoite lysis, presumably by MAC formation in the presence of immune antibodies (Behet et al., 2018; Kurtovic et al., 2018). Moreover, cohort studies in malaria endemic populations have established that *P*. *falciparum* sporozoites are susceptible to antibody-mediated complement fixation and activation, with the circumsporozoite protein (CSP) being an important target of complement fixation (Kurtovic et al., 2018). Additionally, antibodies against *P. falciparum* merozoites can activate the classical pathway, culminating in formation of the MAC and merozoite lysis (Boyle et al., 2015). Results presented here extend previous findings by showing that missense mutations in C5 alter the longitudinal risk for both malaria and SMA.

Selection of the C5 variants for investigation was based on polymorphic variants that could impart amino acid changes, had elevated MAFs in African populations, and were in linkage equilibrium. While both mutations are distal from the C5 convertase cleavage site and are not predicted to have major structural impacts, subtle changes in protein structure may still impact protein function, e.g., by changing the propensity of a molecule to aggregate or by impacting its stability (Goldenzweig and Fleishman, 2018). Since C5b is unstable (Hadders et al., 2012), small changes in stability may result in more rapid degradation or aggregation before binding of other MAC components. Interestingly, the rs17216529:C>T, p. (Val145Ile) variant has previously been non-significantly associated with decreased risk of stroke in pediatric patients with sickle cell disease (Flanagan et al., 2013), and increased levels of the MAC in serum have been associated with incidence and poor outcome of acute ischemic stroke (Si et al., 2019). In Kenyan children, reduced complement lytic activity has been reported in SMA, despite similar levels of C5a between uncomplicated and severe cases (Nyakoe et al., 2009). Thus, although the variants selected for investigation are not predicted to substantially alter the C5 structure, they may impart subtle changes to structure and/or stability of C5b and the MAC which could result in reduced lytic activity.

The main findings that emerged when examining the influence of the C5 variants on longitudinal clinical outcomes was that inheritance of two mutant alleles TT (Ile145Ile) for rs17216529:C>T significantly increased susceptibility to both malaria and SMA. Although SMA is an important cause of childhood mortality in the region, carriage of TT alleles did not significantly alter the risk of all-cause mortality, potentially explaining the relatively high MAF (0.320) maintained in the cohort.

When investigating additional C5 variant combinations, we found that the TC haplotype significantly increased the risk of malaria, consistent with the T allele at rs17216529:C>T being associated with an increased risk of both malaria and SMA in the genotypic models. The only other haplotypic combination that significantly altered longitudinal clinical outcomes was carriage of the CC haplotype that imparted protection against SMA. This result is consistent with both wild type alleles for the two C5 variants being maintained at high proportions in a population residing in a holoendemic *P. falciparum* transmission region.

Consistent with our recent findings showing that C3 variants inducing non-synonymous amino acid changes failed to alter susceptibility to all-cause childhood mortality (Raballah et al., 2021), the C5 variants explored here were also not significantly associated with mortality. These results, however, should not be over interpreted since our efforts over the past 2 decades have dramatically reduced childhood deaths far below what is expected in rural holoendemic *P. falciparum* transmission regions. As such, the influence of the selected C5 variants on all-cause mortality may not be expected to emerge in the 99 deaths that occurred.

In conclusion, while the high frequency variants selected for investigation do not appear to confer structural changes to the protein structure, the two novel mutations in the C5 alter susceptibility to both malaria and SMA. As such, a limitation of the current findings is that the specific mechanism(s) by which the variants impart changes in genetic susceptibility remain unknown. Future investigations on the mechanistic basis of the current findings should foster a deeper understanding on how potential structural and functional changes in C5 alter susceptibility to malaria and SMA during the developmental phase of naturally-acquired malarial immunity.

## Data Availability

The datasets presented in this study can be found in online repositories. The names of the repository/repositories and accession number(s) can be found in the article/Supplementary Material.

## References

[B1] Achieng A. O. Hengartner N. W. Raballah E. Cheng Q. Anyona S. B. Lauve N. (2019). Integrated OMICS platforms identify LAIR1 genetic variants as novel predictors of cross-sectional and longitudinal susceptibility to severe malaria and all-cause mortality in Kenyan children. EBioMedicine 45, 290–302. 10.1016/j.ebiom.2019.06.043 31278068 PMC6642287

[B2] Anyona S. B. Hengartner N. W. Raballah E. Ong'echa J. M. Lauve N. Cheng Q. (2019). Cyclooxygenase-2 haplotypes influence the longitudinal risk of malaria and severe malarial anemia in Kenyan children from a holoendemic transmission region. J. Hum. Genet. 65, 99–113. 10.1038/s10038-019-0692-3 31664161 PMC7255056

[B3] Awandare G. A. Kempaiah P. Ochiel D. O. Piazza P. Keller C. C. Perkins D. J. (2011). Mechanisms of erythropoiesis inhibition by malarial pigment and malaria-induced proinflammatory mediators in an *in vitro* model. Am. J. Hematol. 86, 155–162. 10.1002/ajh.21933 21264897 PMC4703402

[B4] Behet M. C. Kurtovic L. Van Gemert G. J. Haukes C. M. Siebelink-Stoter R. Graumans W. (2018). The complement system contributes to functional antibody-mediated responses induced by immunization with Plasmodium falciparum malaria sporozoites. Infect. Immun. 86, e00920-17. 10.1128/IAI.00920-17 29735521 PMC6013677

[B5] Borg J. Georgitsi M. Aleporou-Marinou V. Kollia P. Patrinos G. P. (2009). Genetic recombination as a major cause of mutagenesis in the human globin gene clusters. Clin. Biochem. 42, 1839–1850. 10.1016/j.clinbiochem.2009.07.014 19631200

[B6] Boyle M. J. Reiling L. Feng G. Langer C. Osier F. H. Aspeling-Jones H. (2015). Human antibodies fix complement to inhibit Plasmodium falciparum invasion of erythrocytes and are associated with protection against malaria. Immunity 42, 580–590. 10.1016/j.immuni.2015.02.012 25786180 PMC4372259

[B7] Cherepnalkovski A. P. Zemunik T. Glamocanin S. Piperkova K. Gunjaca I. Kocheva S. (2015). Molecular characterization of glucose-6-phosphate dehydrogenase deficiency in families from the republic of Macedonia and genotype-phenotype correlation. Med. Arch. 69, 284–288. 10.5455/medarh.2015.69.284-288 26622077 PMC4639331

[B8] Colobran R. Franco-Jarava C. Martin-Nalda A. Baena N. Gabau E. Padilla N. (2016). Novel mutations causing C5 deficiency in three north-african families. J. Clin. Immunol. 36, 388–396. 10.1007/s10875-016-0275-4 27026170

[B9] Davenport O. C. Hittner J. B. Were T. Ouma Y. Ong'echa J. M. Perkins D. J. (2010). Hematological predictors of increased severe anemia in Kenyan children coinfected with Plasmodium falciparum and HIV-1. Am. J. Hematol. 85 (4), 227–233. 10.1002/ajh.21653 20196168 PMC3095458

[B10] Dode C. Krishnamoorthy R. Lamb J. Rochette J. (1993). Rapid analysis of -alpha 3.7 thalassaemia and alpha alpha alpha anti 3.7 triplication by enzymatic amplification analysis. Br. J. Haematol. 83, 105–111. 10.1111/j.1365-2141.1993.tb04639.x 8435317

[B11] Flanagan J. M. Sheehan V. Linder H. Howard T. A. Wang Y. D. Hoppe C. C. (2013). Genetic mapping and exome sequencing identify 2 mutations associated with stroke protection in pediatric patients with sickle cell anemia. Blood 121, 3237–3245. 10.1182/blood-2012-10-464156 23422753 PMC3630835

[B12] Fredslund F. Laursen N. S. Roversi P. Jenner L. Oliveira C. L. Pedersen J. S. (2008). Structure of and influence of a tick complement inhibitor on human complement component 5. Nat. Immunol. 9, 753–760. 10.1038/ni.1625 18536718

[B13] Gaunt T. R. Rodriguez S. Zapata C. Day I. N. (2006). Midas: Software for analysis and visualisation of interallelic disequilibrium between multiallelic markers. BMC Bioinforma. 7, 227. 10.1186/1471-2105-7-227 PMC1479374 16643648

[B14] Githeko A. K. Service M. W. Mbogo C. M. Atieli F. K. Juma F. O. (1994). Origin of blood meals in indoor and outdoor resting malaria vectors in Western Kenya. Acta Trop. 58, 307–316. 10.1016/0001-706x(94)90024-8 7709869

[B15] Goldenzweig A. Fleishman S. J. (2018). Principles of protein stability and their application in computational design. Annu. Rev. Biochem. 87, 105–129. 10.1146/annurev-biochem-062917-012102 29401000

[B16] Greenwood B. M. Brueton M. J. (1974). Complement activation in children with acute malaria. Clin. Exp. Immunol. 18, 267–272. 4619598 PMC1537893

[B17] Hadders M. A. Bubeck D. Roversi P. Hakobyan S. Forneris F. Morgan B. P. (2012). Assembly and regulation of the membrane attack complex based on structures of C5b6 and sC5b9. Cell Rep. 1, 200–207. 10.1016/j.celrep.2012.02.003 22832194 PMC3314296

[B18] Helegbe G. K. Goka B. Q. Kurtzhals J. A. Addae M. M. Ollaga E. Tetteh J. K. (2007). Complement activation in Ghanaian children with severe Plasmodium falciparum malaria. Malar. J. 6, 165. 10.1186/1475-2875-6-165 18086298 PMC2231372

[B19] Henes J. K. Groga-Bada P. Schaeffeler E. Winter S. Hack L. Zdanyte M. (2021). C5 variant rs10985126 is associated with mortality in patients with symptomatic coronary artery disease. Pharmgenomics. Pers. Med. 14, 893–903. 10.2147/PGPM.S307827 34321906 PMC8312322

[B20] International Hap M C. (2003). The international HapMap project. Nature 426, 789–796. 10.1038/nature02168 14685227

[B21] Jumper J. Evans R. Pritzel A. Green T. Figurnov M. Ronneberger O. (2021). Highly accurate protein structure prediction with AlphaFold. Nature 596, 583–589. 10.1038/s41586-021-03819-2 34265844 PMC8371605

[B22] Kilgore K. S. Flory C. M. Miller B. F. Evans V. M. Warren J. S. (1996). The membrane attack complex of complement induces interleukin-8 and monocyte chemoattractant protein-1 secretion from human umbilical vein endothelial cells. Am. J. Pathol. 149, 953–961. 8780399 PMC1865152

[B23] Kisia L. E. Kempaiah P. Anyona S. B. Munde E. O. Achieng A. O. Ong’echa J. M. (2019). Genetic variation in interleukin-7 is associated with a reduced erythropoietic response in Kenyan children infected with Plasmodium falciparum. BMC Med. Genet. 20, 140. 10.1186/s12881-019-0866-z 31420016 PMC6698010

[B24] Klos A. Tenner A. J. Johswich K.-O. Ager R. R. Reis E. S. Köhl J. (2009). The role of the anaphylatoxins in health and disease. Mol. Immunol. 46, 2753–2766. 10.1016/j.molimm.2009.04.027 19477527 PMC2725201

[B25] Kurtovic L. Behet M. C. Feng G. Reiling L. Chelimo K. Dent A. E. (2018). Human antibodies activate complement against Plasmodium falciparum sporozoites, and are associated with protection against malaria in children. BMC Med. 16, 61. 10.1186/s12916-018-1054-2 29706136 PMC5925837

[B26] Lu L. Li Z. Li H. Li X. Vekilov P. G. Karniadakis G. E. (2019). Quantitative prediction of erythrocyte sickling for the development of advanced sickle cell therapies. Sci. Adv. 5, eaax3905. 10.1126/sciadv.aax3905 31457104 PMC6703859

[B27] Machiela M. J. Chanock S. J. (2015). LDlink: A web-based application for exploring population-specific haplotype structure and linking correlated alleles of possible functional variants. Bioinformatics 31, 3555–3557. 10.1093/bioinformatics/btv402 26139635 PMC4626747

[B28] Menny A. Serna M. Boyd C. M. Gardner S. Joseph A. P. Morgan B. P. (2018). CryoEM reveals how the complement membrane attack complex ruptures lipid bilayers. Nat. Commun. 9, 5316. 10.1038/s41467-018-07653-5 30552328 PMC6294249

[B29] Merle N. S. Church S. E. Fremeaux-Bacchi V. Roumenina L. T. (2015). Complement system Part I - molecular mechanisms of activation and regulation. Front. Immunol. 6, 262. 10.3389/fimmu.2015.00262 26082779 PMC4451739

[B30] Morgan B. (2015). The membrane attack complex as an inflammatory trigger. Immunobiology 221, 747–751. 10.1016/j.imbio.2015.04.006 25956457

[B31] Müller-Eberhard H. J. (1985). The killer molecule of complement. J. Invest. Dermatol. 85, 47s–52s. 10.1111/1523-1747.ep12275445 3891882

[B32] Munde E. O. Raballah E. Okeyo W. A. Ong’echa J. M. Perkins D. J. Ouma C. (2017). Haplotype of non-synonymous mutations within IL-23R is associated with susceptibility to severe malaria anemia in a P. falciparum holoendemic transmission area of Kenya. BMC Infect. Dis. 17, 291. 10.1186/s12879-017-2404-y 28427357 PMC5397818

[B33] Novelli E. M. Hittner J. B. Davenport G. C. Ouma C. Were T. Obaro S. (2010). Clinical predictors of severe malarial anaemia in a holoendemic Plasmodium falciparum transmission area. Br. J. Haematol. 149, 711–721. 10.1111/j.1365-2141.2010.08147.x 20408849 PMC3095459

[B34] Nyakoe N. K. Taylor R. P. Makumi J. N. Waitumbi J. N. (2009). Complement consumption in children with Plasmodium falciparum malaria. Malar. J. 8, 7. 10.1186/1475-2875-8-7 19134190 PMC2645421

[B35] Obonyo C. O. Vulule J. Akhwale W. S. Grobbee D. E. (2007). In-hospital morbidity and mortality due to severe malarial anemia in Western Kenya. Am. J. Trop. Med. Hyg. 77, 23–28. 10.4269/ajtmh.77.6.suppl.23 18165471

[B36] Odhiambo C. O. Otieno W. Adhiambo C. Odera M. M. Stoute J. A. (2008). Increased deposition of C3b on red cells with low CR1 and CD55 in a malaria-endemic region of Western Kenya: Implications for the development of severe anemia. BMC Med. 6, 23. 10.1186/1741-7015-6-23 18717995 PMC2562382

[B37] Ong'echa J. M. Keller C. C. Were T. Ouma C. Otieno R. O. Landis-Lewis Z. (2006). Parasitemia, anemia, and malarial anemia in infants and young children in a rural holoendemic *Plasmodium falciparum* transmission area. Am. J. Trop. Med. Hyg. 74, 376–385. 10.4269/ajtmh.2006.74.376 16525094

[B38] Otieno R. O. Ouma C. Ong'echa J. M. Keller C. C. Were T. Waindi E. N. (2006). Increased severe anemia in HIV-1-exposed and HIV-1-positive infants and children during acute malaria. AIDS 20, 275–280. 10.1097/01.aids.0000200533.56490.b7 16511422

[B39] Ouma C. Davenport G. C. Awandare G. A. Keller C. C. Were T. Otieno M. F. (2008). Polymorphic variability in the interleukin (IL)-1beta promoter conditions susceptibility to severe malarial anemia and functional changes in IL-1beta production. J. Infect. Dis. 198, 1219–1226. 10.1086/592055 18781863 PMC2723855

[B40] Pak M. A. Markhieva K. A. Novikova M. S. Petrov D. S. Vorobyev I. S. Maksimova E. S. (2021). Using AlphaFold to predict the impact of single mutations on protein stability and function. bioRxiv, 1–7. 10.1101/2021.09.19.460937 PMC10019719 36928239

[B41] Perkins D. J. Were T. Davenport G. C. Kempaiah P. Hittner J. B. Ong'echa J. M. (2011). Severe malarial anemia: Innate immunity and pathogenesis. Int. J. Biol. Sci. 7, 1427–1442. 10.7150/ijbs.7.1427 22110393 PMC3221949

[B42] Pettersen E. F. Goddard T. D. Huang C. C. Couch G. S. Greenblatt D. M. Meng E. C. (2004). UCSF Chimera-a visualization system for exploratory research and analysis. J. Comput. Chem. 25, 1605–1612. 10.1002/jcc.20084 15264254

[B43] Raballah E. Anyona S. B. Cheng Q. Munde E. O. Hurwitz I. F. Onyango C. (2021). Complement component 3 mutations alter the longitudinal risk of pediatric malaria and severe malarial anemia. Exp. Biol. Med. 247 (8), 672–682. 10.1177/15353702211056272 PMC9039490 34842470

[B44] Ricklin D. Hajishengallis G. Yang K. Lambris J. D. (2010). Complement: A key system for immune surveillance and homeostasis. Nat. Immunol. 11, 785–797. 10.1038/ni.1923 20720586 PMC2924908

[B45] Roestenberg M. Mccall M. Mollnes T. Deuren M. Sprong T. Klasen I. (2007). Complement activation in experimental human malaria infection. Trans. R. Soc. Trop. Med. Hyg. 101, 643–649. 10.1016/j.trstmh.2007.02.023 17481680

[B46] Schaid D. J. Batzler A. J. Jenkins G. D. Hildebrandt M. A. (2006). Exact tests of Hardy-Weinberg equilibrium and homogeneity of disequilibrium across strata. Am. J. Hum. Genet. 79, 1071–1080. 10.1086/510257 17186465 PMC1698709

[B47] Schejbel L. Fadnes D. Permin H. Lappegard K. T. Garred P. Mollnes T. E. (2013). Primary complement C5 deficiencies - molecular characterization and clinical review of two families. Immunobiology 218, 1304–1310. 10.1016/j.imbio.2013.04.021 23743184

[B48] Sherry S. T. Ward M. Sirotkin K. (1999). dbSNP-database for single nucleotide polymorphisms and other classes of minor genetic variation. Genome Res. 9, 677–679. 10.1101/gr.9.8.677 10447503

[B49] Si W. He P. Wang Y. Fu Y. Li X. Lin X. (2019). Complement complex C5b-9 levels are associated with the clinical outcomes of acute ischemic stroke and carotid plaque stability. Transl. Stroke Res. 10, 279–286. 10.1007/s12975-018-0658-3 30173313 PMC6395518

[B50] Team R. C. 2019. R: A language and environment for statistical computing. R Foundation for Statistical Computing, Vienna, Austria: Available at: https://www.R-project.org/ .[Google Scholar].

[B51] Varadi M. Anyango S. Deshpande M. Nair S. Natassia C. Yordanova G. (2022). AlphaFold protein structure database: Massively expanding the structural coverage of protein-sequence space with high-accuracy models. Nucleic Acids Res. 50, D439–D444. 10.1093/nar/gkab1061 34791371 PMC8728224

[B52] Wenisch C. Spitzauer S. Florris-Linau K. Rumpold H. Vannaphan S. Parschalk B. (1997). Complement activation in severe Plasmodium falciparum malaria. Clin. Immunol. Immunopathol. 85, 166–171. 10.1006/clin.1997.4417 9344699

[B53] Were T. Davenport G. C. Hittner J. B. Ouma C. Vulule J. M. Ong'echa J. M. (2011). Bacteremia in Kenyan children presenting with malaria. J. Clin. Microbiol. 49, 671–676. 10.1128/JCM.01864-10 21106789 PMC3043473

[B54] Were T. Hittner J. B. Ouma C. Otieno R. O. Orago A. S. Ong'echa J. M. (2006). Suppression of RANTES in children with Plasmodium falciparum malaria. Haematologica 91, 1396–1399. 17018392 PMC13402947

[B55] WHO (2021). World malaria report. Geneva: World Health Organization. WHO Report.

