# Supplementary material for: Nonsynonymous amino acid changes in the α-chain of complement component 5 influence longitudinal susceptibility to Plasmodium falciparum infections and severe malarial anemia in kenyan children
Source: Front Genet. 2022 Sep 14;13:977810. doi: 10.3389/fgene.2022.977810 (PMC9515573; doi:10.3389/fgene.2022.977810)
Supplement: Supplementary file 1 [file DataSheet1.docx]

**SUPPLEMENTARY MATERIAL**

**Nonsynonymous amino acid changes in the α-chain of complement component 5 influence longitudinal susceptibility to *Plasmodium falciparum* infections and severe malarial anemia in Kenyan children**

Authors: Evans Raballah^1,2^, Kristen Wilding^3^, Samuel B. Anyona^1,4^, Elly O. Munde^1,5^, Ivy Hurwitz^6^, Clinton Onyango^1,7^, Cyrus Ayieko^8^, Christophe G. Lambert^6^, Kristan A. Schneider^9^, Philip D.Seidenberg^10^, Collins Ouma^1,7^, Benjamin H. McMahon^3^, Qiuying Cheng^6^, and Douglas J. Perkins^1,6^

**Table S1**

The distribution of genotypes and haplotypes for rs17216529:C>T, p.(Val151Ile) and rs17610:C>T, p.(Ser1310Asn) in the cohort are shown in **Table S1**. Allele frequencies for rs17216529:C>T, p.(Val151Ile) were: overall population (C=0.680>T=0.320), aparasitemic group, (C=0.670>T=0.330), UM (C=0.690>T=0.310) and SMA (C=0.650>T=0.350). Although there was a significant departure from the Hardy-Weinberg Equilibrium (HWE) in the overall population [Chi-square (χ^2^) =17.855; *P*<0.001], and within each of the three groups: aparasitemic (*P*=0.022), UM (*P*=0.015), and SMA (*P*=0.004), the proportion of rs17216529:C>T genotypes were similar across the study groups (χ^2^ test, *P*=0.234).

Allele frequencies for rs17610:C>T, p.(Ser1310Asn) were: overall population (C=0.890>T=0.110), aparasitemic children (C=0.890>T=0.110), UM (C=0.900>T=0.100) and SMA (C=0.870>T=0.130). Analogous to the allelic distribution displayed by rs17216529:C>T, there was a significant departure from HWE in the overall population (χ^2^=458.347; *P*<0.001) and within each of the three study groups (*P*=<0.001, for all the groups). Consistently, the genotype frequencies for rs17610:C>T were also similar across the groups (χ^2^ test, *P*=0.303).

Frequency distributions of the phased haplotypes from rs17216529:C>T and rs17610:C>T were comparable for the CC (Val151Ser1310, *P*=0.085), CT (Val151Asn1310, *P*=0.812), TC (Ile151Ser1310, *P*=0.671), and TT (Ile151Asn1310, *P*=0.925) combinations.

**Table S1. Distribution of *C5* genotypic and haplotypic variants**

| **Genotype/Haplotype** | **Amino Acid** | **Aparasitemic** | **UM**  **(Hb≥5.0g/dL)** | **SMA**  **(Hb<5.0g/dL)** | **Total** | ***P*** |
| --- | --- | --- | --- | --- | --- | --- |
| **rs17216529:C>T** | **Val151Ile** | n=266 | n=901 | n=261 | n=1,428 |  |
| CC, n (%) | Val151Val | 128 (48.12) | 447 (49.61) | 120 (45.98) | 695 (48.67) |  |
| CT, n (%) | Val151Ile | 101 (37.97) | 353 (39.18) | 98 (37.55) | 552 (38.66) | 0.234 |
| TT, n (%) | Ile151Ile | 37 (13.91) | 101 (11.21) | 43 (16.47) | 181 (12.67) |  |
| Allele Frequency |  | C=0.670 T=0.330 | C=0.690  T=0.310 | C=0.650  T=0.350 | C=0.680  T=0.320 |  |
| HWE |  | χ^2^=5.209  *P*<0.022 | χ^2^=5.895  *P*<0.015 | χ^2^=8.219  *P*<0.004 | χ^2^=17.855  *P*<0.001 |  |
| **rs17610:C>T** | **Ser1310Asn** | n=275 | n=923 | n=275 | n=1473 |  |
| CC, n (%) | Ser1310Ser | 237 (86.18) | 794 (86.02) | 225 (81.82) | 1,256 (85.27)  123 (8.35)  94 (7.48) |  |
| CT, n (%) | Ser1310Asn | 18 (6.55) | 75 (8.13) | 30 (10.91) |  | 0.303 |
| TT, n (%) | Asn1310Asn | 20 (7.27) | 54 (5.85) | 20 (7.27) |  |  |
| Allele Frequency |  | C=0.890  T=0.110 | C=0.900  T=0.100 | C=0.870  T=0.130 | C=0.890  T=0.110 |  |
| HWE |  | χ^2^=117.288  *P*<0.001 | χ^2^=274.219  *P*<0.001 | χ^2^=71.227  *P*<0.001 | χ^2^=458.347  *P*<0.001 |  |
| **rs17216529:C>T/rs17610:C>T** | **Val151Ile/Ser1310Asn** | n=288 | n=971 | n=287 | n=1,546 |  |
| Non-CC, n (%) | non-Val151Ser1310 | 37 (12.85) | 101 (10.40) | 43 (14.98) | 181 (11.71)  1,365 (88.29) | 0.085 |
| CC, n (%) | Val151Ser1310 | 251 (87.15) | 870 (85.60) | 244 (85.02) |  |  |
| Non-CT, n (%) | non-Val151Asn1310 | 155 (53.82) | 534 (55.00) | 152 (52.96) | 841 (54.40) | 0.812 |
| CT, n (%) | Val151Asn1310 | 133 (46.18) | 437 (45.00) | 135 (47.04) | 705 (45.60) |  |
| Non-TC, n (%) | non-Ile151Ser1310 | 260 (90.28) | 883 (90.94) | 256 (89.20) | 1,399 (90.49)  147 (9.51) | 0.671 |
| TC, n (%) | Ile151Ser1310 | 28 (9.72) | 88 (9.06) | 31 (10.80) |  |  |
| Non-TT, n (%) | non-Ile151Asn1310 | 283 (98.26) | 954 (98.25) | 281 (97.91) | 1,518 (98.19) | 0.925 |
| TT, n (%) | Ile151Asn1310 | 5 (1.74) | 17 (1.75) | 6 (2.09) | 28 (1.81) |  |

Data are presented as proportions [n (%)] of genetic variants within the study groups. Study participants were categorized into three groups, aparasitemic (no peripheral blood *P. falciparum* detected), uncomplicated malaria [UM (Hb≥5.0 g/dL with any density). parasitemia)], and severe malarial anemia [SMA (i.e., Hb<5.0 g/dL with any density parasitemia)]. Statistical significance determined by the Chi-square analysis. NB: Percentages for the genotypes and haplotypes are calculated from top to bottom in each column. Note: non-CC refers to non-wild genotypes for both SNPs, consistently non-CT is non-C in the first SNP and non-T in the second SNP.

**Table S2**

Table S2, shows the proportions of HbAS vs. G6PD in the data together with the absolute and relative frequencies of SMA and malaria episodes among the HbAS-G6PD deficient combinations alongside with a chi-square goodness of fit test. As shown in table S2, the SMA episodes are less than expected in children with sickle cell trait and sickle cell disease, while the proportion of children with SMA was higher in children inheriting the “normal” (AA and G6PD normal) genetic traits for both HBAS and G6PD. A similar pattern is observed for malaria episodes. However, in this case, the differences are marginal.

**Table S2: Proportions of HbAS variants and G6PD in the study populations**

| **Genetic variants**  **HbAS/G6PD** | Normal | Intermediate | Deficient |
| --- | --- | --- | --- |
| **Malaria** |  |  |  |
| AA | 4961 (68.08) | 1115 (15.3) | 253 (3.47) |
| AS | 633 (8.69) | 186 (2.55) | 102 (1.4) |
| SS | 24 (0.33) | 6 (0.08) | 7 (0.1) |
|  | χ^2^=50.205 | df=8 | *P*=1.536x10^-8^ |
| **SMA** |  |  |  |
| AA | 313 (76.34) | 44 (10.73) | 14 (3.41) |
| AS | 20 (4.88) | 5 (1.22) | 4 (0.98) |
| SS | 7 (1.71) | 2 (0.49) | 1 (0.24) |
|  | χ^2^=31.437 | df=8 | *P*=1.174x10^-4^ |

Data are presented as proportions [n (%)] of genetic variants combinations. Statistical analyses were performed by χ^2^ goodness of fit test.

**SUPPLEMENTARY FIGURES**


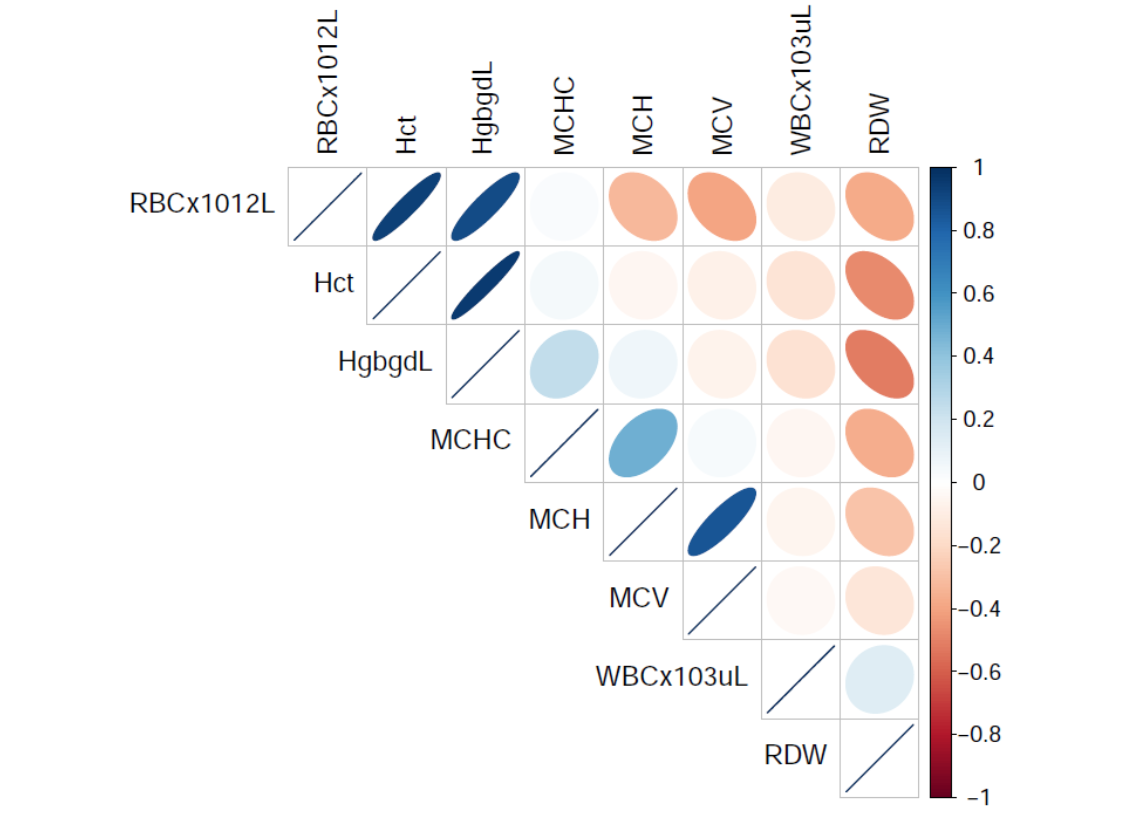


**Supplementary Figure 1**: The correlation between the hematological parameters collected at the time of enrolment. As expected, the red blood cell (RBC) indices such as Hematocrit (Hct), Hemoglobin/dL (HgbdL) and RBC counts were highly correlated. In addition, MCH and MCV were also highly correlated. However, HgbgdL vs MCHC, MCHC vs MCH and WBC vs RDW were weekly correlated.
